# 2-(1*H*-Indol-3-yl)acetohydrazide

**DOI:** 10.1107/S1600536812041694

**Published:** 2012-10-20

**Authors:** Lala Rukh Sidra, Islam Ullah Khan, Muhammad Yar, Jim Simpson

**Affiliations:** a Materials Chemistry Laboratory, Department of Chemistry, GC University, Lahore 54000, Pakistan; b Interdisciplinary Research Center in Biomedical Materials, COMSATS Institute of Information Technology, Lahore 54000, Pakistan; c Department of Chemistry, University of Otago, PO Box 56, Dunedin, New Zealand

## Abstract

In the title compound C_10_H_11_N_3_O, the mean plane of the indole ring system (r.m.s. deviation 0.0131 Å) subtends a dihedral angle of 87.27 (5)° to the almost planar acetohydrazide substituent (r.m.s. deviation 0.0291 Å). In the crystal, bifurcated N—H⋯(O,N) and N—H⋯N hydrogen bonds involving the pyrrole N–H grouping combine to form zigzag chains along *a*. Additional N—H⋯O contacts from the hydrazide N–H group augmented by C—H⋯π inter­actions link the mol­ecules into chains along the *a* axis. The overall effect of these contacts is a three-dimensional network structure with mol­ecules stacked along the *b*-axis direction.

## Related literature

For the use of hydrazides in the synthesis of heterocyclic compounds, see: Narayana *et al.* (2005*a* ▶,*b* ▶) and in the production of pharmaceuticals, see: Liu *et al.* (2006 ▶). For related structures, see: Butcher *et al.* (2007 ▶); Hou (2009 ▶); Li & Ban (2009 ▶); Sarojini *et al.* (2007*a* ▶,*b* ▶,*c* ▶,*d* ▶).
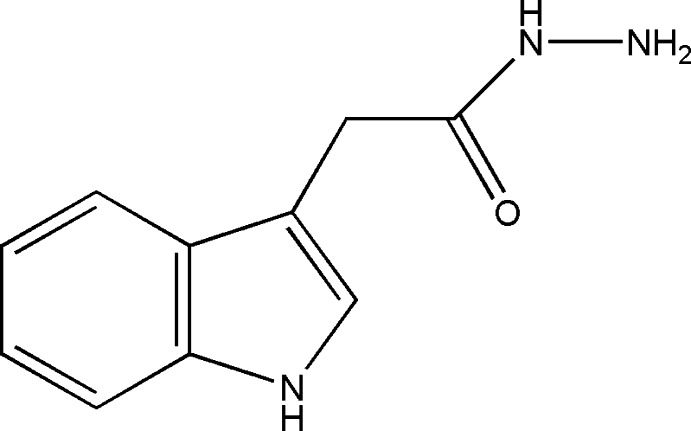


## Experimental

### 

#### Crystal data

C_10_H_11_N_3_O
*M* *_r_* = 189.22
Orthorhombic, 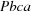

*a* = 12.1599 (7) Å
*b* = 9.6153 (4) Å
*c* = 16.2345 (8) Å
*V* = 1898.16 (16) Å^3^
*Z* = 8
Mo *K*α radiation
μ = 0.09 mm^−1^
*T* = 296 K
0.17 × 0.14 × 0.11 mm

#### Data collection

Bruker APEXII CCD area detector diffractometer
8600 measured reflections
2329 independent reflections
1294 reflections with *I* > 2σ(*I*)
*R*_int_ = 0.039

#### Refinement

*R*[*F* ^2^ > 2σ(*F* ^2^)] = 0.046
*wR*(*F* ^2^) = 0.122
*S* = 1.00
2329 reflections
139 parameters
H atoms treated by a mixture of independent and constrained refinement
Δρ_max_ = 0.16 e Å^−3^
Δρ_min_ = −0.16 e Å^−3^

### 

Data collection: *APEX2* (Bruker 2005 ▶); cell refinement: *APEX2* and *SAINT* (Bruker 2005 ▶); data reduction: *SAINT*; program(s) used to solve structure: *SHELXS97* (Sheldrick, 2008 ▶); program(s) used to refine structure: *SHELXL97* (Sheldrick, 2008 ▶); molecular graphics: *ORTEP-3* (Farrugia, 1997 ▶) and *Mercury* (Macrae *et al.*, 2008 ▶); software used to prepare material for publication: *SHELXL97*, *enCIFer* (Allen *et al.*, 2004 ▶), *PLATON* (Spek, 2009 ▶), *publCIF* (Westrip 2010 ▶).

## Supplementary Material

Crystal structure: contains datablock(s) global, I. DOI: 10.1107/S1600536812041694/hg5253sup1.cif

Structure factors: contains datablock(s) I. DOI: 10.1107/S1600536812041694/hg5253Isup2.hkl

Supplementary material file. DOI: 10.1107/S1600536812041694/hg5253Isup3.cml

Additional supplementary materials:  crystallographic information; 3D view; checkCIF report

## Figures and Tables

**Table 1:** Hydrogen-bond geometry (Å, °) *Cg*2 is the centroid of the C1–C6 benzene ring.

*D*—H⋯*A*	*D*—H	H⋯*A*	*D*⋯*A*	*D*—H⋯*A*
N1—H1*N*⋯O10^i^	0.80 (2)	2.21 (2)	2.927 (2)	149.4 (19)
N1—H1*N*⋯N3^i^	0.80 (2)	2.50 (2)	3.126 (2)	136.6 (19)
N2—H2*N*⋯O10^ii^	0.89 (2)	2.20 (2)	3.0799 (19)	166.3 (17)
C9—H9*A*⋯*Cg*2^iii^	0.97	2.73	3.644 (2)	157

Symmetry codes: (i) 
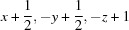
; (ii) 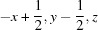; (iii) 
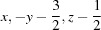
.

**Fig. 1.**
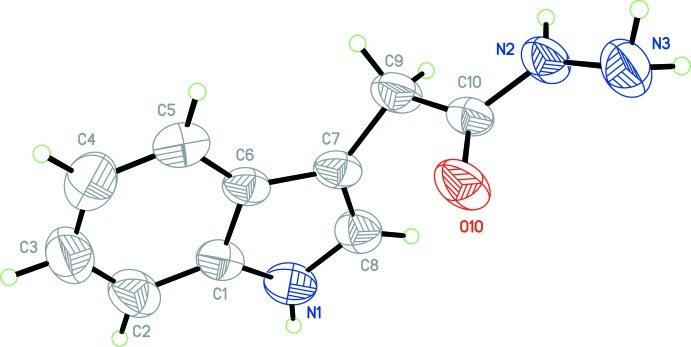
The structure of the title compound showing the atom numbering scheme with displacement ellipsoids drawn at the 50% probability level

**Fig. 2.**
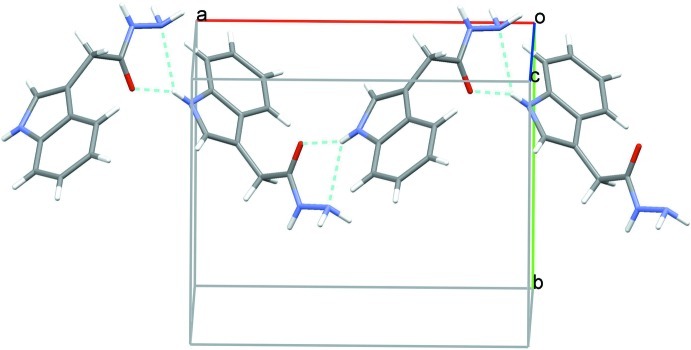
Zigzag chains along a with hydrogen bonds drawn as dashed lines.

**Fig. 3.**
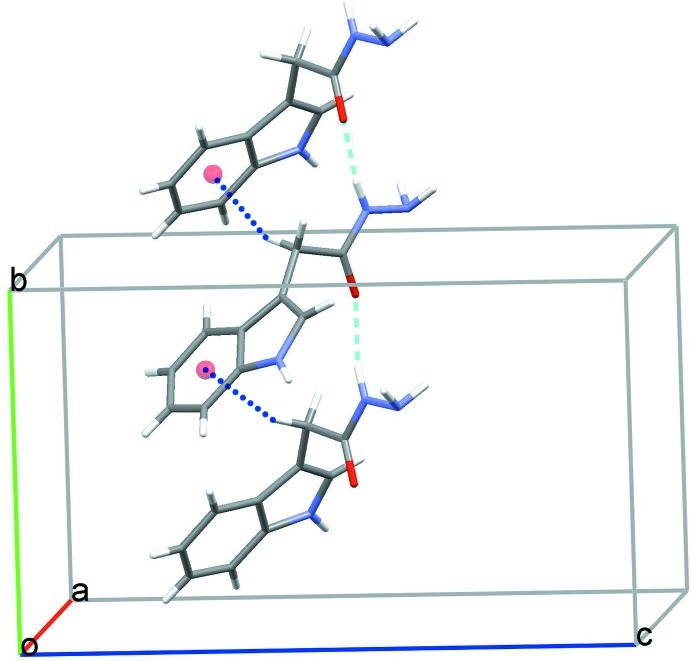
Molecules linked into rows along the b by N–H···O hydrogen bonds (dashed lines) and C–H···π contacts (dotted lines).

**Fig. 4.**
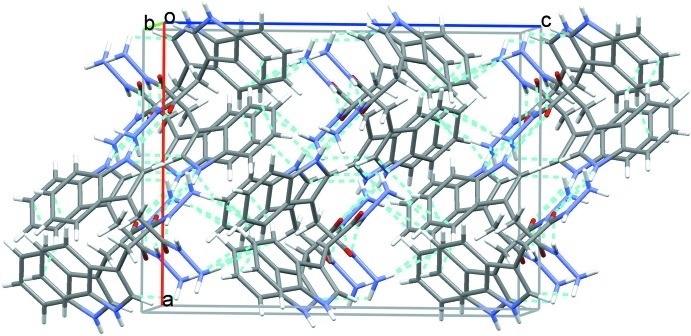
A three dimensional network structure of molecules stacked along the b axis with hydrogen bonds drawn as dashed lines.

## supplementary crystallographic information

### Comment

Hydrazides are useful precursors in the synthesis of several heterocyclic compounds. (Narayana *et al.*, 2005*a*,*b*). They are also intermediates in the production of many pharmaceutically important compounds (Liu *et al.*, 2006). The structures of a number of hydrazides and their derivatives have also been reported (Butcher *et al.*, 2007; Hou, 2009; Li & Ban, 2009; Sarojini *et al.*, 2007*a*,*b*,*c*,*d*).

In the title hydrazide compound, the indole ring system is planar (r.m.s. deviation 0.0131 Å) and subtends an angle of 87.27 (5)° to the C9, C10, O10, N2, N3 acetohydrazide substituent which is also planar (r.m.s. deviation 0.0291 Å). In the crystal structure, bifurcated N1–H1N···O10 and N1–H1N···N6 hydrogen bonds together form zigzag chains along *a*, Table 1, Fig 2. Additional N2–H2N···O10 contacts augmented by C9–H9A···π interactions link the molecules into rows along *b*, Fig 3. The overall effect of these contacts is a three dimensional network structure with molecules stacked along the *b* axis, Fig 4.

### Experimental

Indole 3-methyl ester (500 mg, 2.6 mmole, 1eq) was added to hydrazine hydrate (80%, 4eq) in ethanol. The reaction mixture was refluxed for 2–3 h, allowed to cool and poured into 100 ml of chilled water. The resulting solid was filtered, dried and re-crystallized from ethanol to obtain the product (300 mg, 60%), mp: 143°C. The purity of the compound was confirmed using thin layer chromatography Rf: 0.18, (n-hexane: ethyl acetate). Crystals of the title compound suitable for X-ray analysis were grown from a solution in ethanol at room temperature.

### Refinement

N bound H atoms were located in difference Fourier maps and their coordinates were refined with *U*_iso_=1.2*U*_eq_ (N). All H-atoms bound to carbon were refined using a riding model with d(C—H) = 0.93 Å, for aromatic and 0.97 Å for CH_2_ H atoms with *U*_iso_ = 1.2*U*_eq_ (C).

### Crystal data

**Table:**

C_10_H_11_N_3_O	*F*(000) = 800
*M**_r_* = 189.22	*D*_x_ = 1.324 Mg m^−^^3^
Orthorhombic, *P**b**c**a*	Mo *K*α radiation, λ = 0.71073 Å
Hall symbol: -P 2ac 2ab	Cell parameters from 1251 reflections
*a* = 12.1599 (7) Å	θ = 3.0–22.1°
*b* = 9.6153 (4) Å	µ = 0.09 mm^−^^1^
*c* = 16.2345 (8) Å	*T* = 296 K
*V* = 1898.16 (16) Å^3^	Prism, colorless
*Z* = 8	0.17 × 0.14 × 0.11 mm

### Data collection

**Table:**

Bruker APEXII CCD area detector diffractometer	1294 reflections with *I* > 2σ(*I*)
Radiation source: fine-focus sealed tube	*R*_int_ = 0.039
Graphite monochromator	θ_max_ = 28.3°, θ_min_ = 3.0°
φ and ω scans	*h* = −16→14
8600 measured reflections	*k* = −12→12
2329 independent reflections	*l* = −20→21

### Refinement

**Table:**

Refinement on *F*^2^	Primary atom site location: structure-invariant direct methods
Least-squares matrix: full	Secondary atom site location: difference Fourier map
*R*[*F*^2^ > 2σ(*F*^2^)] = 0.046	Hydrogen site location: inferred from neighbouring sites
*wR*(*F*^2^) = 0.122	H atoms treated by a mixture of independent and constrained refinement
*S* = 1.00	*w* = 1/[σ^2^(*F*_o_^2^) + (0.0512*P*)^2^ + 0.1536*P*] where *P* = (*F*_o_^2^ + 2*F*_c_^2^)/3
2329 reflections	(Δ/σ)_max_ < 0.001
139 parameters	Δρ_max_ = 0.16 e Å^−^^3^
0 restraints	Δρ_min_ = −0.16 e Å^−^^3^

### Special details

**Table:**

Geometry. All e.s.d.'s (except the e.s.d. in the dihedral angle between two l.s. planes) are estimated using the full covariance matrix. The cell e.s.d.'s are taken into account individually in the estimation of e.s.d.'s in distances, angles and torsion angles; correlations between e.s.d.'s in cell parameters are only used when they are defined by crystal symmetry. An approximate (isotropic) treatment of cell e.s.d.'s is used for estimating e.s.d.'s involving l.s. planes.
Refinement. Refinement of *F*^2^ against ALL reflections. The weighted *R*-factor *wR* and goodness of fit *S* are based on *F*^2^, conventional *R*-factors *R* are based on *F*, with *F* set to zero for negative *F*^2^. The threshold expression of *F*^2^ > σ(*F*^2^) is used only for calculating *R*-factors(gt) *etc*. and is not relevant to the choice of reflections for refinement. *R*-factors based on *F*^2^ are statistically about twice as large as those based on *F*, and *R*- factors based on ALL data will be even larger.

### Fractional atomic coordinates and isotropic or equivalent isotropic displacement parameters (Å^2^)

**Table:**

	*x*	*y*	*z*	*U*_iso_*/*U*_eq_	
N1	0.51122 (13)	0.27482 (16)	0.60889 (11)	0.0549 (5)	
H1N	0.5591 (17)	0.321 (2)	0.5893 (12)	0.066*	
C1	0.45025 (15)	0.30809 (17)	0.67668 (12)	0.0463 (5)	
C2	0.46525 (18)	0.4106 (2)	0.73576 (14)	0.0615 (6)	
H2	0.5251	0.4707	0.7335	0.074*	
C3	0.3892 (2)	0.4202 (2)	0.79725 (14)	0.0683 (6)	
H3	0.3980	0.4878	0.8377	0.082*	
C4	0.29916 (19)	0.3318 (2)	0.80094 (13)	0.0664 (6)	
H4	0.2479	0.3428	0.8430	0.080*	
C5	0.28434 (16)	0.22822 (19)	0.74347 (12)	0.0542 (5)	
H5	0.2242	0.1687	0.7466	0.065*	
C6	0.36154 (14)	0.21440 (16)	0.68030 (11)	0.0418 (4)	
C7	0.37296 (14)	0.12255 (16)	0.61199 (11)	0.0437 (4)	
C8	0.46342 (15)	0.16439 (18)	0.57039 (12)	0.0512 (5)	
H8	0.4894	0.1235	0.5222	0.061*	
C9	0.29943 (16)	0.00197 (17)	0.59225 (12)	0.0526 (5)	
H9A	0.2600	−0.0243	0.6418	0.063*	
H9B	0.3448	−0.0764	0.5762	0.063*	
C10	0.21718 (14)	0.02925 (16)	0.52500 (11)	0.0404 (4)	
O10	0.18296 (11)	0.14649 (11)	0.50855 (8)	0.0575 (4)	
N2	0.18364 (13)	−0.08386 (15)	0.48613 (10)	0.0505 (4)	
H2N	0.2143 (16)	−0.165 (2)	0.4996 (11)	0.061*	
N3	0.09921 (17)	−0.07433 (16)	0.42677 (12)	0.0631 (5)	
H3N1	0.1226 (17)	−0.128 (2)	0.3824 (13)	0.076*	
H3N2	0.0381 (19)	−0.125 (2)	0.4406 (14)	0.076*	

### Atomic displacement parameters (Å^2^)

**Table:**

	*U*^11^	*U*^22^	*U*^33^	*U*^12^	*U*^13^	*U*^23^
N1	0.0422 (9)	0.0513 (10)	0.0711 (12)	−0.0088 (7)	0.0042 (9)	0.0060 (9)
C1	0.0431 (10)	0.0411 (9)	0.0548 (11)	−0.0031 (7)	−0.0091 (9)	0.0057 (9)
C2	0.0620 (14)	0.0526 (11)	0.0699 (15)	−0.0112 (10)	−0.0161 (12)	−0.0005 (10)
C3	0.0867 (17)	0.0570 (13)	0.0612 (14)	0.0031 (12)	−0.0161 (13)	−0.0101 (11)
C4	0.0741 (15)	0.0708 (14)	0.0542 (13)	0.0138 (12)	0.0036 (11)	0.0026 (11)
C5	0.0498 (11)	0.0527 (11)	0.0600 (12)	−0.0022 (9)	0.0011 (10)	0.0136 (10)
C6	0.0415 (9)	0.0341 (8)	0.0497 (10)	0.0006 (7)	−0.0065 (8)	0.0085 (8)
C7	0.0442 (10)	0.0335 (8)	0.0534 (11)	0.0010 (7)	−0.0056 (9)	0.0087 (8)
C8	0.0537 (11)	0.0422 (10)	0.0577 (12)	0.0064 (8)	0.0021 (10)	0.0009 (9)
C9	0.0625 (12)	0.0328 (9)	0.0624 (12)	−0.0039 (8)	−0.0095 (10)	0.0070 (8)
C10	0.0444 (10)	0.0282 (8)	0.0485 (10)	−0.0012 (7)	0.0029 (8)	0.0015 (7)
O10	0.0671 (9)	0.0300 (6)	0.0752 (9)	0.0049 (6)	−0.0198 (7)	−0.0020 (6)
N2	0.0627 (10)	0.0292 (8)	0.0594 (10)	0.0020 (7)	−0.0114 (9)	−0.0020 (7)
N3	0.0782 (13)	0.0455 (10)	0.0656 (12)	−0.0004 (8)	−0.0177 (11)	−0.0082 (8)

### Geometric parameters (Å, º)

**Table:**

N1—C8	1.362 (2)	C6—C7	1.425 (2)
N1—C1	1.365 (3)	C7—C8	1.352 (2)
N1—H1N	0.80 (2)	C7—C9	1.499 (2)
C1—C2	1.387 (3)	C8—H8	0.9300
C1—C6	1.407 (2)	C9—C10	1.504 (2)
C2—C3	1.364 (3)	C9—H9A	0.9700
C2—H2	0.9300	C9—H9B	0.9700
C3—C4	1.388 (3)	C10—O10	1.2310 (18)
C3—H3	0.9300	C10—N2	1.322 (2)
C4—C5	1.377 (3)	N2—N3	1.411 (2)
C4—H4	0.9300	N2—H2N	0.89 (2)
C5—C6	1.397 (2)	N3—H3N1	0.93 (2)
C5—H5	0.9300	N3—H3N2	0.92 (2)
			
C8—N1—C1	108.72 (15)	C8—C7—C6	106.48 (15)
C8—N1—H1N	124.2 (15)	C8—C7—C9	127.50 (18)
C1—N1—H1N	125.9 (15)	C6—C7—C9	126.01 (16)
N1—C1—C2	130.74 (18)	C7—C8—N1	110.48 (17)
N1—C1—C6	107.46 (16)	C7—C8—H8	124.8
C2—C1—C6	121.79 (18)	N1—C8—H8	124.8
C3—C2—C1	117.75 (19)	C7—C9—C10	114.65 (14)
C3—C2—H2	121.1	C7—C9—H9A	108.6
C1—C2—H2	121.1	C10—C9—H9A	108.6
C2—C3—C4	121.66 (19)	C7—C9—H9B	108.6
C2—C3—H3	119.2	C10—C9—H9B	108.6
C4—C3—H3	119.2	H9A—C9—H9B	107.6
C5—C4—C3	121.2 (2)	O10—C10—N2	123.07 (16)
C5—C4—H4	119.4	O10—C10—C9	122.82 (15)
C3—C4—H4	119.4	N2—C10—C9	114.10 (14)
C4—C5—C6	118.58 (18)	C10—N2—N3	119.81 (15)
C4—C5—H5	120.7	C10—N2—H2N	118.4 (12)
C6—C5—H5	120.7	N3—N2—H2N	121.8 (12)
C5—C6—C1	119.03 (17)	N2—N3—H3N1	105.6 (13)
C5—C6—C7	134.12 (16)	N2—N3—H3N2	112.8 (15)
C1—C6—C7	106.84 (16)	H3N1—N3—H3N2	97.9 (18)
			
C8—N1—C1—C2	179.29 (19)	C5—C6—C7—C8	177.68 (18)
C8—N1—C1—C6	0.1 (2)	C1—C6—C7—C8	−1.15 (18)
N1—C1—C2—C3	179.47 (19)	C5—C6—C7—C9	−3.4 (3)
C6—C1—C2—C3	−1.5 (3)	C1—C6—C7—C9	177.72 (16)
C1—C2—C3—C4	−0.5 (3)	C6—C7—C8—N1	1.3 (2)
C2—C3—C4—C5	1.6 (3)	C9—C7—C8—N1	−177.58 (16)
C3—C4—C5—C6	−0.7 (3)	C1—N1—C8—C7	−0.9 (2)
C4—C5—C6—C1	−1.2 (2)	C8—C7—C9—C10	−79.8 (2)
C4—C5—C6—C7	−179.92 (18)	C6—C7—C9—C10	101.5 (2)
N1—C1—C6—C5	−178.42 (15)	C7—C9—C10—O10	−26.0 (3)
C2—C1—C6—C5	2.3 (3)	C7—C9—C10—N2	155.06 (17)
N1—C1—C6—C7	0.62 (18)	O10—C10—N2—N3	−4.6 (3)
C2—C1—C6—C7	−178.61 (16)	C9—C10—N2—N3	174.30 (17)

### Hydrogen-bond geometry (Å, º)

Cg2 is the centroid of the C1–C6 benzene ring.

**Table:**

*D*—H···*A*	*D*—H	H···*A*	*D*···*A*	*D*—H···*A*
N1—H1*N*···O10^i^	0.80 (2)	2.21 (2)	2.927 (2)	149.4 (19)
N1—H1*N*···N3^i^	0.80 (2)	2.50 (2)	3.126 (2)	136.6 (19)
N2—H2*N*···O10^ii^	0.89 (2)	2.20 (2)	3.0799 (19)	166.3 (17)
C9—H9*A*···*Cg*2^iii^	0.97	2.73	3.644 (2)	157

Symmetry codes: (i) *x*+1/2, −*y*+1/2, −*z*+1; (ii) −*x*+1/2, *y*−1/2, *z*; (iii) *x*, −*y*−3/2, *z*−1/2.
